# The impact of integrated care on quality of life in community-dwelling older adults with frailty: A systematic review and mixed method synthesis

**DOI:** 10.1016/j.tjfa.2026.100150

**Published:** 2026-06-02

**Authors:** Joanne Boyce, Declan Patton, Chanel Watson, Tom O’Connor, Zena Moore, Linda Nugent

**Affiliations:** a Community RGN, HSE, Ireland; b School of Nursing and Midwifery, Royal College of Surgeons in Ireland, Dublin, Ireland

**Keywords:** Integrated care, Frailty, Older adults, Quality of life, Community-dwelling

## Abstract

**Background:**

Frailty among older adults is an escalating public health challenge, often associated with poorer quality of life (QoL) and increased pressure on healthcare systems. Integrated care is proposed as a strategy to meet the complex needs of this population, though evidence of its effectiveness remains inconclusive.

**Objectives:**

To determine the impact of integrated care on the quality of life of frail, community-dwelling older adults.

**Design:**

Systematic review and mixed-methods synthesis, including meta-analysis and narrative synthesis.

**Setting:**

Community-based healthcare systems across six international studies.

**Participants:**

A total of 5498 frail, community-dwelling older adults across six studies: four randomised controlled trials and two quasi-experimental designs.

**Intervention:**

Integrated care interventions tailored to frailty, including person-centred, multidisciplinary, and value-based models.

**Measurements:**

Quality of Life was the primary outcome. Secondary outcomes included social functioning and healthcare costs. Standardised tools such as SF-12, SF-36, ICECAP-O, and EQ-5D were used across studies.

**Results:**

The meta-analysis showed a small, non-significant improvement in QoL (SMD = 0.13, 95 % CI: -0.09 to 0.35, *p* = 0.24) with high heterogeneity (I² = 91 %). Tailored, multidimensional models showed greater effects, particularly in preserving social functioning. Findings on cost-effectiveness were inconsistent; some studies reported reduced hospital use, while others found increased primary care visits without cost savings.

**Conclusions:**

. Integrated care **may support social functioning** but does not demonstrate a consistent improvement in overall QoL. Future trials should standardise QoL measurement, report intervention components clearly, include economic evaluations, and assess longer-term outcomes.

## 1 Introduction & background

The global ageing population is steadily increasing, which, according to [1], reflects the positive impact of advancements in public health and social welfare that have enabled people to live longer lives. However, the authors also highlight the ongoing challenges older individuals encounter in accessing appropriate health and social care services. The World Health Organization (WHO) [2] estimates that by 2050, the percentage of people aged 60 and over will nearly double—from 12 % to 22 %. This demographic shift is anticipated to place considerable pressure on healthcare systems, complicating the delivery of high-quality, cost-effective care to this expanding group [[3], [4]] also highlight the fact that the growing number of older adults, many of whom experience physical and cognitive decline, presents significant obstacles for healthcare services. The rising incidence of frailty within this population intensifies these challenges, further increasing the burden on healthcare systems and resources [5].Frailty is a dynamic condition that can improve or worsen over time rather than a fixed state, meaning individuals may move between stages of robustness, pre-frailty, and frailty [6]. It typically progresses through three stages: robustness, pre-frailty, and frailty, with individuals capable of moving back and forth between these phases [7]. While robust older adults may face health setbacks, these are often manageable and can allow them to return to previous levels of functioning [8]. It is important to differentiate between frailty and ageing, as [6] point out that advanced age does not automatically lead to the adverse outcomes commonly linked with frailty.

Although there is no single, universally accepted definition of frailty, it is generally recognised as an age-related syndrome marked by a decline in several physiological systems, leaving individuals more susceptible to deteriorating health and poor outcomes [9]. Frailty is associated with a greater risk of falls, hospitalisation, disability, and mortality [10,11]. As the population continues to age, the incidence of frailty is expected to rise substantially, reinforcing the need for integrated care strategies within primary care settings [12]. Supporting this view, Cergi et al., [13] advocate for early identification and intervention through community-based care, arguing that comprehensive assessments and person-centred care models can enhance outcomes for those living with frailty.

International estimates suggest that frailty affects around one in ten adults aged over 65 years, with prevalence rising substantially with advanced age, particularly among those over 85 [14]. Reported prevalence varies considerably depending on how frailty is operationalised, most commonly through the frailty phenotype or the frailty index/deficit accumulation approach, as well as multidomain instruments that include psychosocial components [14]. In Ireland, frailty prevalence has been estimated at 12.7 % among adults aged over 50 years and 21.5 % among those over 65 years [8]. A systematic review by [14] also explored the relationship between frailty and Quality of Life (QoL), noting growing interest but mixed findings.

Frailty often limits independence by hindering the ability to perform everyday tasks, thereby negatively impacting QoL [15]. The World Health Organization [16] defines QoL as an individual’s perception of their life within the context of cultural norms, personal goals, and expectations.

As frailty becomes a more pressing concern, there is growing recognition that health systems must evolve to address the complex needs of older adults living with this condition [10] describe frailty as an emerging public health issue that demands systemic change to meet the needs of an ageing population. Traditional healthcare models, which are largely disease-focused, are often ill-equipped to manage the multifaceted nature of frailty, leading to fragmented and inadequate care [6,17] argue that once frailty is identified, care must be reoriented to centre around the individual and be coordinated through primary care, using a multidisciplinary approach. Supporting this direction, the WHO [18] developed the Integrated Care for Older People (ICOPE) approach, which promotes a coordinated and person-centred model of care tailored to the complex needs of ageing populations. ICOPE aims to maintain and enhance older adults’ intrinsic capacity and functional ability by ensuring continuity of care across health and social services. As part of the United Nations Decade of Healthy Ageing (2021–2030), the implementation of ICOPE within Universal Health Coverage is recognised as a global priority for advancing more sustainable and inclusive health systems [19,20]. Hendy et al., [21] assert that integrated care is key to preventing further decline in frail individuals [4] support this, showing that shifting care from institutional settings to community-based services can significantly improve health outcomes. Their three-year study demonstrated reductions in emergency department visits and hospitalisations [22], drawing on data from The Irish Longitudinal Study on Ageing (TILDA), emphasise the importance of integrated care in enabling older adults to access appropriate services and remain living in their homes. For this review, “integrated care” was defined broadly as coordinated, person-centred, multidisciplinary care designed to address the complex health and social needs of frail older adults. While the WHO’s Integrated Care for Older People (ICOPE) framework (WHO, 2025) represents a key example, included studies were not limited to ICOPE based models and encompassed a range of integrated care approaches implemented in community settings.

Quality of life (QoL) is a key outcome in frailty research, reflecting not only physical health but also psychological, social, and functional well-being [16]. It is widely regarded as a patient-centred measure that captures the broader impact of interventions beyond clinical outcomes. The concept of a minimally important difference (MID) is often used to interpret changes in QoL, representing the smallest change perceived as meaningful by patients [23]. Although MIDs were not consistently reported in included studies, recognising this concept is important for evaluating whether observed changes are clinically significant. Understanding the impact of integrated care on QoL is therefore critical for informing both care delivery and health policy for frail older adults. In addition to QoL, this review examined social functioning and healthcare costs as secondary outcomes. Frailty often leads to reduced social participation and isolation, which are strongly linked to poorer mental health and reduced QoL [24]. Likewise, frailty is associated with higher healthcare utilisation and costs, making cost outcomes highly relevant to policymakers and service planners seeking sustainable care models (Clegg et al., 2013)

Frailty significantly impacts health systems worldwide, increasing the need for effective, patient-centred care models that support older adults. While there is substantial literature on frailty assessment, screening tools, and intervention strategies, there is a notable lack of research specifically addressing how integrated care improves QoL for community-dwelling older adults with frailty. This evidence gap makes it difficult to identify the most effective strategies for enhancing well-being and preserving independence in this vulnerable group. As such, conducting a systematic review is crucial to consolidate existing knowledge, highlight best practices, and develop evidence-based recommendations for improving integrated care approaches aimed at supporting frail older adults.

### 1.1 Aim and research question

The objective of this systematic review is to determine whether integrated care affects Quality of Life (QoL) in community-dwelling older adults. Future recommendations grounded in evidence for education, research, and policy can be derived from the findings of this systematic review.

Clinical questions can be structured using a four-part model developed by Richardson et al. (1995), known as PICO, which aids in formulating research questions and conducting literature searches [25]. This systematic review applies the PICO framework as follows:

What is the impact of Integrated Care on Quality of Life in Community-dwelling older adults with frailty?

Population: Community-dwelling older adults with frailty

Intervention: Integrated Care

Comparison: Usual Care

Outcome: Quality of Life

### 1.2 Outcomes measured

The primary outcome of the present systematic review was Quality of Life

The secondary outcomes of this systematic review were:

• Social Functioning
• Healthcare Costs

### 1.3 Search strategy

The present systematic review included studies that met the following criteria, the inclusion criteria were:

• Studies focusing on integrated care.
• Studies involving community-dwelling older adults with frailty
• Studies reporting on Quality of Life
• Studies that were available in the English language

The exclusion criteria were:

• Studies involving adults living in community nursing units/ nursing homes
• Studies in a foreign language
• Studies that did not consider primary or secondary outcome

Search methods

Both randomised controlled trials (RCTs) and quasi-experimental (non-randomised) studies were eligible. No restrictions were applied regarding duration of intervention or follow-up. A review protocol was not formally registered; however, the methodology was guided by PRISMA 2020 standards to ensure transparency and replicability.

A structured search was developed using Medical Subject Headings (MeSH) and free-text terms related to integrated care, frailty, older adults, and quality of life. Searches were conducted in CINAHL, MEDLINE, PsycINFO, and EMBASE between December 2024 and January 2025. The search strategy was adapted for each database to ensure sensitivity and specificity.

An example of the full search string used in CINAHL (EBSCOhost) is presented below to support replicability:

(frail* OR "frailty syndrome" OR "frail elderly" OR "frail older adult*" OR "geriatric frailty") AND ("integrated care" OR "coordinated care" OR "multidisciplinary care" OR "person-centred care" OR "collaborative care" OR "care integration") AND ("quality of life" OR QoL OR "health-related quality of life" OR HRQoL OR wellbeing OR "life satisfaction") AND ("community-dwelling" OR "home-based" OR "primary care" OR "community care")

### 1.4 Data extraction

Data extraction was performed independently by two reviewers using a structured data extraction form to capture key study information, including design, population characteristics, intervention details, outcomes, and findings relevant to the review question. Discrepancies were resolved through discussion and consensus. The PRISMA 2020 flow diagram was used to document the study selection process and illustrate the number of records identified, screened, and included in the final synthesis (Page et al., 2020)

### 1.5 Data analysis and synthesis

This systematic review employs a mixed-methods approach to assess the impact of integrated care on Quality of Life (QoL) in frail older adults. A random-effects meta-analysis was conducted using Rev Man to quantify the overall effect of interventions, while a narrative synthesis was used to explore heterogeneity and contextual differences between studies. A mixed-methods approach was selected to enable both statistical synthesis of quantitative data and a deeper exploration of the contextual factors surrounding the interventions. This dual approach is especially useful when assessing complex interventions such as integrated care, which can influence a range of outcomes that may not be fully represented through quantitative measures alone. A random-effects meta-analysis model assumes that variations in treatment effect across studies arise not only from random sampling differences but also from genuine differences in treatment effects due to variations in study populations, interventions, or settings [26].

## 2 Quality appraisal

The methodological quality of all included studies was assessed using the Evidence-Based Librarianship (EBL) Critical Appraisal Checklist [27]. Although originally developed for evaluating evidence quality in library and information science, this tool has been applied across health and social science research because of its comprehensive structure and adaptability to diverse study designs. The EBL checklist assesses four domains population, data collection, study design, and results allowing for consistent comparison across both randomised and non-randomised studies. Each study was independently appraised by two reviewers, and discrepancies were resolved through discussion. Studies scoring ≥75 % were considered valid, while those below thithreshold were interpreted with caution.

Results of the appraisal are summarised in Table 4

## 3 Results

Following the removal of duplicates, 51 abstracts were screened, and 15 full-text articles were assessed for eligibility. Six studies met the inclusion criteria, comprising a total of 5498 frail, community-dwelling older adults. The included studies consisted of four randomised controlled trials [[28], [29], [30], [31]] and two quasi-experimental designs [32,33]. These were conducted across diverse settings, including the Netherlands (*n* = 3), Spain (*n* = 1), Taiwan (*n* = 1), and Argentina (*n* = 1). All studies evaluated integrated care interventions for community-dwelling older adults with frailty, though inclusion criteria, intervention components, and outcome measures varied (Table 1)

**Table 1:** Characteristics of included studies.

Author (Year), Country	Population (Mean Age; % Female; Frailty Instrument)	Intervention (Components, Delivery, Intensity/Duration, and Setting)	Comparator	Quality-of-Life Instrument(s)	Secondary Outcomes	Study Design / Sample Size
[28], Netherlands	Community-dwelling adults aged ≥75 years (n = 3092); mean age = 74.2 years (SD 8.4); 55.2 % female; frailty identified using EMR-based U-PRIM system and Groningen Frailty Indicator (GFI, cut-off ≥ 4).	Three-step proactive, nurse-led primary-care programme: (1) Frailty identification for patients at risk; (2) Practice-nurse-led comprehensive geriatric assessment (CGA) at home covering ten health domains; (3) Tailor-made care plan developed collaboratively by the nurse and GP with evidence-based interventions. Follow-up home visits and telephone contacts were provided at 6 and 12 months.	Usual GP care	SF-12 (6 and 12 months)	GP consultations, hospitalisations	Cluster RCT (59 GP practices; n = 3092)
[30], Netherlands	Community-dwelling adults aged ≥65 years (n = 1147); mean age = 80.5 years (SD 7.5); 66.5 % female; frailty identified by PRISMA-7 ≥ 3.	Geriatric Care Model (GCM): Practice nurses performed multidimensional RAI-CHA home assessments every six months. Tailored care plans were developed with GPs. Complex cases were discussed in multidisciplinary meetings with geriatric teams. Supervision and training were provided by expert geriatricians. Follow-up at 6, 12, 18, and 24 months.	Usual primary care	SF-36 (baseline, 6, 12, 18, 24 months)	Social functioning, instrumental ADL, cost-effectiveness	Stepped-wedge cluster RCT; n = 1147
[32], Netherlands	Community-dwelling adults aged ≥70 years (n = 377); mean age = 82.0 years; 65 % female; frailty identified with Groningen Frailty Indicator (GFI ≥ 4; baseline mean 6.0 ± 2.0).	Walcheren Integrated Care Model (WICM): Proactive frailty screening and needs assessment using EASYcare. Each participant received a personalised care plan coordinated by a GP and case manager. Multidisciplinary teams (GPs, geriatricians, nurses, social workers) met regularly. Duration: 12 months; assessments at 3 and 12 months.	Usual GP-led care	ICECAP-O; EQ-5D	Social well-being, cost-utility	Quasi-experimental; n = 377
[33], Argentina	Community-dwelling frail older adults aged ≥70 years (n = 242); frailty defined by clinical judgement and social/functional vulnerability (polypharmacy, falls, ADL dependence).	Health and Social Care Integration Programme: Each participant had a health–social-care counsellor supervised by a medical coordinator. Comprehensive home-based assessments informed a person-centred plan. Follow-up home visits and phone contacts occurred throughout the 12-month period, with multidisciplinary case reviews.	Usual care (no integrated coordination)	Local validated QoL scale (baseline and 12 months)	Hospital admission risk, mortality	Quasi-experimental; n = 242
[31], Taiwan	Community-dwelling older adults aged ≥65 years with ≥3 chronic conditions (n = 398); mean age = 73.2 years (SD 6.3); 60 % female; high risk of decline (prefrail/frail phenotype).	TIGER multidomain integrated-care programme: 12-month multidisciplinary intervention covering physical exercise, cognitive training, nutritional counselling, medication review, vascular-risk management, and social engagement. Delivered through 16 × 2-hour sessions per year in community centres. Framework aligned with WHO ICOPE and ICHOM standards.	Usual primary care	SF-36 (baseline, 3, 6, 9, 12 months)	Cognitive performance, healthcare utilisation	Pragmatic RCT; n = 398
[29], Spain	Community-dwelling adults aged ≥65 years (n = 240); frailty measured using Tilburg Frailty Index + UCLA-3 Loneliness Scale.	Value-Based Integrated Care (VALUECARE): ICT-enabled, person-centred model. Baseline assessment followed by co-designed personalised care plan targeting social participation and mental well-being. Plans monitored via ValueCare App and Vida24 platform. Monthly reviews for 12 months, with outcomes at 12 and 18 months.	Usual primary care	ICHOM Standard Set for Older People (PROMs)	Social participation, hospitalisations, service use	Pre–post controlled trial; n = 240

## 4 Study designs

The six studies in this systematic review use varied research designs to evaluate integrated care interventions for frail older adults. Four studies were Randomised Controlled Trials (RCTs): [30] conducted a stepped wedge cluster RCT on a geriatric care model, while [28] and [31] implemented cluster RCTs assessing proactive care models and multidomain interventions [29] used an RCT with a pre-post evaluation design to examine a personalised frailty intervention. RCTs minimize bias through randomisation, providing a rigorous method for evaluating cause-and-effect relationships [34]. Two studies, [33] and [32], used quasi-experimental designs. These studies assessed intervention-outcome relationships without random assignment, allowing for impact evaluation while acknowledging potential bias [35]

### 4.1 Primary outcome

The results of the random-effects meta-analysis indicate that integrated care interventions did not have a statistically significant effect on Quality of Life (QoL) among frail, community-dwelling older adults. The pooled effect size (SMD = 0.13, 95 % CI: −0.09 to 0.35, *p* = 0.24) crosses zero, indicating no significant difference between intervention and control groups. High heterogeneity (I² = 91 %) suggests substantial variation between studies. Five of the six included studies reported no statistically significant improvement in QoL, while one study, [33] showed a significant positive effect (SMD = 0.80, *p* = 0.001). However, this was a non-randomised study, which limits the strength of this finding [29] reported a positive trend (SMD = 0.20) that did not reach statistical significance in pooled analysis. Differences in the type, intensity, and duration of interventions may have influenced outcomes, as well as variations in QoL instruments used across studies. While measures such as the SF-12 and SF-36 primarily reflect physical and mental health, tools like ICECAP-O and ICHOM include broader domains such as capability and social participation, potentially affecting sensitivity to change.

QoL was assessed using varied instruments across studies: SF-12 (Bleijenberg), SF-36 (Hoogendijk), ICECAP-O and EQ-5D (Looman), ICHOM Standard Set (Fernández-Salido), WHOQOL-BREF (Wei-Ju), and locally adapted measures (Perman). To enable pooled analysis, results were converted to standardised mean differences (SMDs). Although these instruments capture different QoL dimensions (physical, mental, social), SMD pooling is appropriate when constructs are conceptually related [36]. These differences in measurement tools may influence how changes in QoL are detected and reported, as some instruments are more sensitive to specific aspects of integrated care interventions, such as mental well-being or social functioning. As such, inconsistencies in outcome measures may contribute to the mixed findings seen across studies and highlight the need for greater standardisation in QoL assessment within future research.

Only Perman et al. demonstrated a statistically significant positive effect (SMD = 0.80, *p* = 0.001). Fernández-Salido et al. reported a positive trend (SMD = 0.20), though the 95 % CI crossed zero in pooled analysis, indicating non-significance.Two studies, Hoogendijk et al.,. (2016) and [28], reported no significant improvements in QoL, with effect sizes of −0.02 and 0.01, respectively, and non-significant p-values (0.45 and 0.78). These findings suggest that frailty screening and general geriatric care models alone may have limited impact on overall QoL [31] also found a significant improvement in mental health-related QoL (*p* = 0.019), though physical QoL improvements were less pronounced. Some studies showed improvements in targeted QoL domains rather than overall QoL. For instance, [32] found that while general QoL scores were not significantly affected, participants in the intervention group experienced preserved social well-being (effect size = 0.15, *p* = 0.04), a dimension that declined in the control group.

### 4.2 Secondary outcome

#### 4.2.1 Social functioning

Social functioning was a key secondary outcome in several studies [32] reported significantly preserved social well-being over 12 months (SMD = 0.15, *p* = 0.04), while [29] found improved social engagement/participation (SMD = 0.20, *p* = 0.002) [30] found no significant difference between groups (SMD = −0.02, 95 % CI: −0.10 to 0.06, *p* = 0.45)In contrast, [32] reported that participants in the integrated care group maintained (“preserved”) their social well-being over the 12-month period (SMD = 0.15, 95 % CI: 0.05 to 0.25, *p* = 0.04), whereas the control group experienced a significant decline. Therefore, the observed effect reflects a significant preservation rather than an overall improvement in social well-being Similarly, [29] found a statistically significant improvement in social participation and engagement among intervention participants (SMD = 0.20, 95 % CI: 0.10 to 0.30, *p* = 0.002).

Together, these findings suggest that while integrated care did not consistently improve overall Quality of Life, it may have significant benefits in maintaining or enhancing social functioning, an important component of well-being for frail older adults (Table 2).

**Table 2:** Social functioning outcomes by study.

Study	Definition of Social Functioning	Instrument Used	Effect Size (SMD)	95 % CI	p-value	Finding
[30]	Social functioning subscale (participation in social activities)	SF-36	−0.02	−0.10, 0.06	0.45	No significant Difference
[32]	Social well-being (capability to connect with others)	ICECAP-O	0.15	0.05, 0.25	0.04	Significantly preserved Intervention group. Control group showed decline
[29]	Social participation and engagement	ICHOM Standard Set	0.20	0.10, 0.30	0.002	Significantly Improved

NOTE: Forest plot includes both randomised and quasi-experimental studies [32] and [33] were non-randomised and should be interpreted with caution.

### 4.3 Healthcare costs

Healthcare cost and utilisation outcomes were variably reported across studies, with differing metrics and levels of economic evaluation [28] found that participants receiving proactive nurse-led care had more GP consultations and home visits than those in usual care (Mean Rate = 9.34 vs. 7.12 per year, *p* = 0.002), but there was no reduction in hospital or emergency visits [32] conducted an economic evaluation alongside their quasi-experimental study, estimating an incremental cost-effectiveness ratio (ICER) of approximately €35,000 per Quality-Adjusted Life Year (QALY) gained, indicating that integrated care improved well-being at modest additional cost rather than generating direct savings [33] reported a significant reduction in hospital admission risk among participants receiving integrated care (HR = 0.503, 95 % CI: 0.340–0.746, *p* = 0.001), suggesting potential downstream cost benefits. In contrast, [30] found no difference in hospitalisation-free survival, defined as the time from baseline to the first hospitalisation or death, between intervention and control groups (*p* > 0.05) [31] presented limited economic data, noting only minor changes in healthcare utilisation patterns without associated cost analysis. Finally, [29] reported that their value-based integrated care model significantly reduced healthcare resource use and hospitalisations (*p* < 0.05), suggesting improved system efficiency. Overall, these findings indicate inconsistent evidence regarding the cost impact of integrated care for frail older adults. While some studies demonstrated reduced hospital use or improved efficiency, others found increased primary care engagement or limited cost data, highlighting the need for more robust economic evaluations (Table 3).

**Table 3:** Healthcare cost findings.

Study	Cost Indicator(s)	Key Findings	Interpretation
[28]	GP consultations, home visits, hospital admissions	Intervention group had significantly more GP consultations and home visits (Mean Rate = 9.34 vs. 7.12 per year, p = 0.002), but no reduction in hospital or emergency visits.	Increased primary care use without clear cost savings.
[32]	Cost-effectiveness (incremental cost per capability gain)	Economic evaluation using EQ-5D and ICECAP-O indicated improved well-being at slightly higher cost, with an incremental cost-effectiveness ratio (ICER) of €35,000 per QALY.	Integrated care may enhance well-being but was not cost-saving within one year.
[33]	Hospital admission risk	Intervention group had significantly lower hospital admission risk (HR = 0.503, 95 % CI: 0.340–0.746, p = 0.001).	Suggests potential reduction in secondary care costs.
[30]	Hospitalisation-free survival (time to first hospitalisation or death)	No significant difference between groups over 24 months (p > 0.05).	Integrated care did not extend hospitalisation-free survival.
[31]	Healthcare utilisation (primary, outpatient, inpatient)	Descriptive reporting only; slight reduction in outpatient visits but no cost data or statistical analysis provided.	Cost impact not assessed quantitatively.
[29]	Healthcare resource use and hospitalisations	Value-based care group showed significant reduction in hospitalisations and resource use (p < 0.05).	Suggests improved efficiency and potential cost savings.

**Table 4:** Quality appraisal.

The validity of Included Studies					
Author	Category result %				Overall result
	Population	Data Collection	Study Design	Results	
[28],	100 %	87.5 %	100 %	100 %	96.3 %
[30]	88.8 %	87.5 %	100 %	100 %	92.8 %
[32]	77.7 %	71.4 %	80 %	83.3 %	77.7 %
[33]	87.5 %	62.5 %	100 %	100 %	85.1 %
[31]	100 %	87.5 %	100 %	100 %	96.3 %
[29]	100 %	75 %	100 %	100 %	92.5 %

NOTE: Quality appraisal conducted using the Evidence-Based Librarianship (EBL) Critical Appraisal Checklist [27], which evaluates four domains: population, data collection, study design, and results. Studies scoring ≥75 % were considered valid.

### 4.4 Methodological quality of included studies

Although all six studies met the inclusion criteria, their methodological quality varied. Randomised controlled trials (Bleijenberg, Hoogendijk, Wei-Ju, and Fernández-Salido) achieved higher validity scores (≥90 %), while the quasi-experimental studies by [32] and [33] scored lower (77.7 % and 85.1 %, respectively), reflecting greater potential for bias due to the absence of randomisation. These studies were therefore interpreted with appropriate caution when considering their contribution to overall conclusions.

## 5 Discussion

This systematic review found no statistically significant effect of integrated care interventions on Quality of Life (QoL) among frail, community-dwelling older adults. While one non-randomised study [33] reported a significant improvement, the overall pooled result did not demonstrate a meaningful change in QoL. The pooled result of the meta-analysis showed no statistically significant improvement in QoL (SMD = 0.13, 95 % CI: −0.09 to 0.35, *p* = 0.24), with high heterogeneity (I² = 91 %) across studies. Among the six included studies, only [33] demonstrated a statistically significant improvement in QoL (SMD = 0.80, *p* = 0.001). Although Fernández-Salido et al. reported a positive effect (SMD = 0.20), the confidence interval crossed zero in the pooled analysis, indicating non-significance [33] study involved intensive, personalised case management delivered over 12 months by a multidisciplinary team, which may have contributed to its positive outcome. Although one non-randomised study [33] reported an improvement in QoL, the remaining studies did not show statistically significant effects. This makes it challenging to identify whether differences in outcomes were influenced by the type, intensity, or duration of the integrated care intervention. While some models were described as “standardised” and others as “tailored,” these approaches often overlap in practice. For example, [28] implemented a structured, evidence-informed framework that was simultaneously adapted to individual patient needs through a comprehensive geriatric assessment. This demonstrates that standardised pathways can still allow for personalisation, and therefore should not be viewed as entirely separate or contrasting models of care.

The magnitude of improvement in QoL across studies appeared to vary, but the mechanisms underlying these differences could not be clearly established from the available data. Although some studies reported enhanced social well-being and engagement, these findings were inconsistent and often drawn from secondary analyses. Consequently, it is not possible to conclude which specific components of integrated care (e.g., coordination, continuity, or multidisciplinary input) contributed most to these outcomes. A more nuanced understanding of how individual intervention components influence quality of life would require process evaluations alongside outcome measurement.

Furthermore, interpreting the impact of integrated care within randomised trial designs in primary care settings presents challenges. As elements of comprehensive geriatric assessment (CGA) and multidisciplinary coordination have increasingly become embedded in routine primary care, the distinction between “usual care” and “integrated care” has narrowed. This may result in a cohort effect, whereby control groups also receive aspects of integrated or coordinated care, thus diminishing observable between-group differences [21]. Future evaluations may require pragmatic or hybrid designs to better capture the real-world impact of such complex, system-level interventions.

The review also highlights important findings related to secondary outcomes, particularly social functioning. In several studies, integrated care interventions supported the maintenance or improvement of social engagement. Even where overall QoL did not improve, social functioning remained stable or showed some gains. Social functioning, measured using tools such as ICECAP-O, ICHOM, and SF-36, was preserved or improved in some intervention groups despite minimal changes in overall QoL. This suggests that integrated care may support specific dimensions of well-being, particularly social participation and engagement, even when global QoL scores remain unchanged This implies that integrated care can have targeted benefits, even if broader QoL indicators remain unchanged [[7], [24]].

QoL is a complex and multifaceted concept, particularly when applied to older adults with frailty [[6], [24]]. It encompasses a range of domains from physical, emotional, psychological, and social and vary in relevance depending on individual preferences, cultural values, and health status [14,37]. Standardised QoL tools, while useful for cross-study comparison, may fail to fully capture the lived experiences of older adults, particularly when interventions affect less evident areas such as autonomy, social connectedness, or emotional resilience [23,38]. In addition, QoL scores can be influenced by personal expectations, adaptation to illness, and environmental context, making consistent interpretation challenging [14]. The varied use of measurement tools across studies (e.g., SF-36, WHOQOL-BREF, ICECAP-O) likely contributed to inconsistent findings, as each tool emphasises different QoL domains. While the use of standardised mean differences allowed for pooling, differences in instrument sensitivity and focus may have influenced effect sizes. Studies that showed greater improvements in QoL or social functioning typically involved multidisciplinary teams, person-centred care plans, and longer follow-up durations (e.g., [33]). In contrast, models focused solely on screening or single-discipline care were less effective.

To address these limitations, complementary outcome measures should be considered alongside traditional QoL assessments. Alternatives such as patient satisfaction, functional independence, and goal attainment scaling offer more personalised insights into the effectiveness of integrated care [39]. For example, goal attainment allows patients to identify and measure progress towards what matters most to them, while functional measures track day-to-day abilities that are often directly impacted by frailty [40,41]. However, these tools also have their limitations, goal attainment may lack standardisation, and satisfaction scores can be affected by expectations rather than actual health improvement [[40], [41], [42]]. Therefore, a combination of outcome measures may offer a more comprehensive understanding of the real-world impact of integrated care on older adults’ well-being.

In terms of healthcare costs, the evidence remains mixed. Some studies reported reduced hospitalisation risk (Perman et al.) or fewer admissions (Fernández-Salido), while others (e.g., Bleijenberg) found increased use of primary care services. However, most studies did not account for the cost of delivering the integrated care interventions themselves. This limits conclusions about cost-effectiveness, as higher primary care engagement may offset downstream savings. Future evaluations should incorporate total intervention costs to assess economic value more comprehensively. Some studies showed reduced hospital use or optimised resource allocation, while others found no significant changes in cost or utilisation patterns. In some cases, increased primary care use was observed, which could reflect a shift toward more preventative, community-based care. However, this did not always result in cost savings or reduced admissions. These mixed findings suggest that integrated care may be more effective at changing how care is delivered rather than reducing overall costs [4,21,22] Variation in reported cost outcomes may reflect differences in healthcare funding models, measurement methods (e.g., hospitalisations vs. primary care usage), and follow-up periods. Some studies captured only direct utilisation, while others included broader economic indicators.

The methodological quality of included studies varied, with randomised controlled trials achieving higher validity scores than quasi-experimental designs. While all studies were included in the synthesis, those with lower quality were interpreted cautiously due to potential risks of bias. Despite this variation, the consistency of certain findings such as improvements in social well-being and individualised care benefits adds weight to the overall conclusions.

This review has certain limitations, including the exclusion of non-English studies, which may have introduced language bias. The relatively small number of eligible studies limits the breadth of the evidence base. Differences in outcome measures and intervention components also reduced comparability [43]. Additionally, heterogeneity across studies was high, reducing the certainty of pooled estimates. The substantial heterogeneity observed likely reflects the genuine diversity of integrated care approaches across international contexts, underscoring the complexity of this field and the continuing need to synthesise such varied evidence. While subgroup analysis was not conducted due to the small number of included studies, future research should explore heterogeneity through stratified analyses by intervention type, care setting, or frailty severity to identify which models yield the most benefit.

To strengthen future evidence, there is a need for greater standardisation in QoL measurement tools and intervention reporting [6]. Future research should focus on pragmatic and implementation-oriented evaluations, using population-level and routinely collected data where appropriate, while ensuring adequate follow-up and clear reporting of intervention components [22].Including economic evaluations would also help determine whether integrated care models are not only clinically beneficial but also cost-effective [22]. Exploring the impact of specific intervention elements, such as coordination, continuity, and person-centred care, may clarify which components contribute most to improved outcomes [44]

Evidence from related studies supports the potential value of integrated, multidisciplinary approaches for older adults. For example, [45] reported that community-based integrated care programmes improved functional independence and reduced hospital admissions, while Sánchez-Rodríguez et al. [46] observed improvements in patient-reported well-being and continuity of care among frail older adults. However, these outcomes reflect the benefits of well-established practices that are increasingly characteristic of contemporary primary care rather than entirely novel interventions. This suggests that the incremental benefit of additional integration may now be smaller, underscoring the need for future studies to evaluate sustainability, scalability, and cost-effectiveness rather than efficacy alone.

## 6 Conclusion

Integrated care has the potential to support frail, community-dwelling older adults, yet its overall impact on Quality of Life (QoL) remains uncertain. Across studies, no significant pooled improvement in QoL was observed, though individual findings suggest potential benefits for social functioning and mental well-being in some contexts. These inconsistencies likely reflect variation in intervention design, implementation intensity, and participant characteristics, as well as the diversity of QoL instruments used. Given the multidimensional nature of QoL, its measurement presents an ongoing challenge in assessing complex, person-centred interventions. Future research should extend beyond conventional QoL metrics to include complementary outcomes such as patient-reported goal attainment, satisfaction, and functional independence, which may more accurately capture the benefits of integrated care. Greater standardisation in outcome measures and more transparent reporting of intervention components would enhance comparability and synthesis across studies.

In practice, integrated care models that are multidisciplinary, person-centred, and responsive to the broader determinants of health remain the most promising. However, the growing incorporation of comprehensive geriatric assessment and coordinated care within routine practice may make it increasingly difficult to demonstrate incremental effects in trial settings. Sustained investment in workforce development, coordination infrastructure, and interprofessional collaboration is needed to embed and optimise these approaches. Further high-quality, longitudinal evaluations will be essential to clarify their long-term clinical, social, and economic impact for an ageing population.

## Fundings (ou funding sources)

This work was supported by the HSE Nursing & Midwifery Planning and Development Unit (NMPDU) Midlands.

Declaration of the use of generative AI and AI-assisted technologies in scientific writing and in figures, images and artwork

**Fig. 1:**
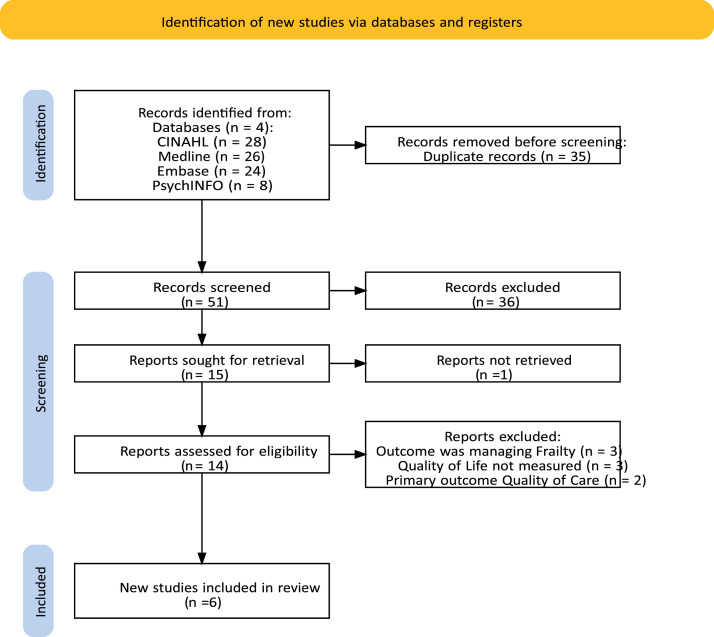
Prisma Flow Diagram. From: Page MJ, McKenzie JE, Bossuyt PM, Boutron I, Hoffmann TC, Mulrow CD, et al. The PRISMA 2020 statement: an updated guideline for reporting systematic reviews. BMJ 2021;372:n71. doi: 10.1136/bmj.n71.

The authors did not use generative AI or AI-assisted technologies in the writing of this article (Fig. 1).

## Ethical statement

This was a systematic review the privacy rights of human subjects have been observed and that informed consent was obtained for experimentation with human subjects in the studies included in this review.

Informed consent was not necessary for this article as it was a systematic review.

- Approval ± registration number

N/A

## Data statement

PRISMA reporting guidelines were followed for this systematic review. Links to datasets are not available as this was a systematic review.

## CRediT authorship contribution statement

**Joanne Boyce:** Writing – original draft, Software, Resources, Methodology, Investigation, Data curation, Conceptualization. **Declan Patton:** Writing – review & editing. **Chanel Watson:** Supervision. **Tom O’Connor:** Supervision. **Zena Moore:** Supervision. **Linda Nugent:** Writing – review & editing, Validation, Supervision.

## Conflict of interest

On behalf of all authors, the corresponding author states that there is no conflict of interest.
